# Food intake and cardiometabolic risk factors in rural Uganda

**DOI:** 10.1186/s13690-021-00547-x

**Published:** 2021-02-25

**Authors:** Therese L. F. Holmager, Dan W. Meyrowitsch, Silver Bahendeka, Jannie Nielsen

**Affiliations:** 1 Centre for Epidemiological Research, Nykøbing Falster Hospital, University of Copenhagen, Ejegodvej 63, DK-4800 Nykøbing Falster, Denmark; 2 Global Health Section, Department of Public Health, University of Copenhagen, Copenhagen, Denmark; 3 Mother Kevin Post Graduate Medical School, Uganda Martyrs University, Kampala, Uganda; 4 Hubert Department of Global Health, Rollins School of Global Health, Emory University, Atlanta, GA USA

**Keywords:** Type 2 diabetes, Overweight, Cardiometabolic, Diet, Sub-Saharan Africa

## Abstract

**Background:**

Along with a nutritional transition in Sub-Saharan Africa, the prevalence of non-communicable diseases is increasing rapidly. We assess the association between food intake and cardiometabolic risk factors in a rural population in Uganda.

**Methods:**

The present study was based on data from a household-based case-control study of diabetic and non-diabetic households in Southwestern Uganda, 2012–2013. We analysed food intake in 359 individuals age ≥ 13 years from 87 households, using a household food frequency questionnaire, and measures of glycated haemoglobin (HbA1c), height and weight. We used multinomial logistic regression to model abnormal HbA1c (≥5.7%) and weight status (underweight, normal weight and overweight) as an outcome of total food intake and by nine food groups. Results were reported as odds ratios (OR) with 95% confidence intervals (CI). Models were adjusted for three nested sets of covariates.

**Results:**

The diet primarily consisted of staple food (cassava and plantain). High-Glycaemic Index staple food was the most consumed food group (median = 14 servings/week, p25-p75: 11–17). Milk, meat, fish and vegetables were the least consumed food groups (medians: 0–3 servings/week). Median intake of sugary food was 6 servings/week (p25-p75: 2–9). The OR of having abnormal HbA1c or being overweight increased with every weekly serving of food (1.02, 95% CI: 1.00–1.04 and 1.01 95% CI: 1.00–1.03, respectively). Of specific food groups, each weekly serving of meat increased the OR of being overweight with 33% (95% CI: 1.08–1.64), and fruit intake decreased the OR of abnormal HbA1c (0.94, 95% CI: 0.88–1.00), though this latter association was attenuated after adjustment for weight status, aerobic capacity, and socioeconomic status.

**Conclusion:**

Diet was monotonous, mainly consisting of cassava and plantain, and increasing food intake was associated with abnormal HbA1c and overweight. To prevent non-communicable diseases a diet with higher intake of fish and vegetables, and less sugary food is recommended.

**Supplementary Information:**

The online version contains supplementary material available at 10.1186/s13690-021-00547-x.

## Background

Low- and middle income countries (LMIC) are experiencing a nutritional transition characterized by traditional diets being replaced by energy-dense and processed food and beverages [1]. In Sub-Sahara Africa (SSA), these dietary changes are occurring along with an epidemiological transition characterized by a change in the burden of diseases from primarily infectious diseases like tuberculosis and malaria to non-communicable diseases (NCDs) such as cardiometabolic conditions like type 2 diabetes (T2D) and obesity [2, 3].

Historically, the diet in SSA consisted of fruits, green leafy vegetables, legumes and whole grain [4]; food items associated with a lower risk of developing cardiometabolic conditions [5], and included in the healthy diet recommendations from the World Health Organization (WHO) [6]. In contrast, the nutritional transition is exposing LMIC populations to food items such as red/processed meat, refined grains, fat and sugary drinks, which have been associated with increased risk of cardiometabolic conditions [5]. Despite a rapidly increasing burden of cardiometabolic conditions in SSA only few studies have investigated the association between diet and cardiometabolic conditions in this region. A Kenyan cross-sectional study identified several dietary risk factors for NCDs in the population; 13.7% always added sugar to beverages and 94% ate less than 5 servings of fruits/vegetables a day [7].

In Uganda, the diet typically includes plantain, maize, cassava, millet, yams, peanuts, pumpkin, beans, green vegetables, and different fruits, supplemented by smaller amounts of meat and fish [8]. However, rice, wheat bread, sweet potatoes, meat and soda have become a greater part of the diet [8]. In Uganda, in 2014 the prevalence of diabetes was 1.4% [9]. However, the International Diabetes Federation predicts that the number of people living with diabetes in Sub-Saharan African (SSA) will increase with 156% between 2017 and 2045 [10]. In Uganda, data from the Demographic and Health Survey found that from 1995 to 2016 the number of overweight women increased from 9.8 to 16.2% and the number of obese women from 2.0 to 6.2% [11]. Further, a study from eastern Uganda found that in 2013 17.8% of the population was overweight and 7% were obese, though overweight was higher among women than men and higher among people living in urban than rural areas [12].

The effect of food intake on cardiometabolic risk factors has been heavily investigated in high income countries (HIC). However, the increasing prevalence of cardiometabolic conditions in SSA [5, 10] where food items and health systems differ from HIC, highlights a need for research in SSA populations.

The aim of the present study was to investigate food intake and its association with cardiometabolic risk factors in a rural Ugandan population potentially being in the midst of a nutritional transition.

## Methods

### Study population

We conducted cross-sectional observational analyses using data from a case-control household study in Kasese District, Southwestern Uganda in 2012–2013, with cases being households including a member diagnosed with T2D and controls being households without any members with diagnosed T2D [13]. From hospital files, we identified people with T2D and invited them and all people age ≥ 13 years in their household to participate in the study. Control households were selected using a random sampling plan (Additional file 1). Initially 100 households were invited to the study and 90 of these households (45 cases and 45 controls) participated. Response rate of individuals living in the 90 households was 97.5%. The case-control study included a total of 437 participants aged ≥13 years. Households were excluded if one or more household member reported that they had HIV/AIDS, other forms of diabetes (e.g. type 1 diabetes), active tuberculosis, severe mental illness, alcoholism, or drug addiction. Details are described elsewhere [13]. In the present study, we also excluded participants diagnosed with T2D (*n* = 45), self-reported malaria (*n* = 4), sickle cell disease (*n* = 1) and those being pregnant (*n* = 12) as these conditions (or treatment) may influence the association between food intake and glycated haemoglobin (HbA1c) and weight status. Furthermore, we excluded 12 participants due to missing information about food consumption and 4 participants due to missing information about aerobic capacity. This resulted in an analytical sample of 359 participants.

### Variables

HbA1c (%), height (cm) and weight (kg) and estimated aerobic capacity (VO_2_-max) [14] was measured for each participant. We used structured questionnaire information about age, sex, household food consumption, household socioeconomic status (SES), years of education, alcohol intake and smoking. Measures and questionnaire data were collected by trained personnel in the local language, Lukonjo. Details are described elsewhere [13].

We categorised food intake according to the Food and Agriculture Organization of the United Nations’ (FAO) food groups [15], but in contrast to FAO, we did not distinguish between different types of vegetables and fruits. Furthermore, the food groups; eggs, organ meat, oils and fat were not included in the food frequency questionnaire (FFQ) used in the present study. We divided FAO’s staple food group into two groups according to Foster-Powell et al.’s International Table of Glycaemic Index (GI) using a cut-off of 70 for low- vs. high-GI [16]. This resulted in nine food groups; high-GI staple food, low-GI staple food, legumes, meat, milk, fish, sugary food, fruits and vegetables (Additional file 2). Consumption of each food group was calculated as the sum of total servings per week.

We used HbA1c as a proxy for diabetes risk. The American Diabetes Association recommends cut-off level for diagnosing diabetes at HbA1c ≥ 6.5%, while HbA1c ≥ 5.7 is considered prediabetes [17]. In the present study population, HbA1c ≥ 5.7 was used as the cut-off level abnormal HbA1c.

Body mass index (BMI), calculated as weight(kg)/height(m)^2^ was used to define weight status for adults while we used WHO’s sex and age specific BMI z-score (BMIZ) for participants aged ≤19 years [18]. Weight status was categorised as; underweight (BMI < 18.5 or BMIZ<-1 SD), normal weight (18.5 ≤ BMI < 25 or − 1 < BMIZ<+ 1 SD) and overweight (BMI ≥ 25 or BMIZ> + 1 SD) [18, 19]. Due to a low number of obese participants (*n* = 8), overweight and obese were merged into one group.

We included aerobic capacity and household SES as co-variates. Aerobic capacity was categorised into three groups (low, medium and high) according to Astrand [20]. Household SES was determined using principal component analysis [21] to score SES at household level and create a categorical variable with three groups – low, medium and high (tertiles). Individuals living in the same household were assigned to the same SES group.

### Statistical analysis

Characteristics of the study participants were reported as proportions or means with 95% confidence intervals (CI) for the total study population and by sex. Food intake was visualized for the total study population as boxplots including median, p25, p75, minimum and maximum values for the nine food groups. To model HbA1c or weight status as a function of total food intake and separate food groups, we used multinomial logistic regression with household as a random effect to account for household clustering. All models were adjusted for sex and age (Model I). As a next step, we adjusted for total food intake (Model II) and lastly we ran a model (Model III) also adjusting for aerobic capacity. In the analysis of HbA1c, weight status was included as a co-variate in Model III. The results were presented as odds ratios (OR) with 95% CI. The analyses were conducted using STATA (14.2, Texas, USA).

## Results

### Characteristics of study participants

Overall, the study population included 57.9% women, mean age was 35.5 years (95% CI: 33.4–37.6) and 41.5% of the participants shared household with a person with T2D (Table 1). The mean HbA1c level was 5.4 (95% CI: 5.4–5.5) and 25.9% of the participants had abnormal HbA1c. Of the participants, 44 (12.3%), 52 (14.5%) and 263 (73.3%) were underweight, overweight and normal weight, respectively. Majority of the participants had a medium aerobic capacity (43.2%).

**Table 1:** Characteristics of study population (≥13 years of age) in Kasese District, Uganda (*N* = 359), Food Intake and Cardiometabolic Risk Factors in Rural Uganda study, 2012–2013

Characteristic	Total	Men	Women
Sex, n (%)			
Men	151 (42.1)	151 (100)	–
Women	208 (57.9)	–	208 (100)
Age in years, mean (95% CI)	35.5 (33.4–37.6)	33.5 (30.1–36.8)	37.0 (34.3–39.7)
Years of education, mean (95% CI)	5.3 (4.9–5.7)	6.0 (5.5–6.6)	4.75 (4.2–5.3)
SES-tertile, n (%)			
Low	116 (32.3)	54 (35.8)	62 (29.8)
Medium	129 (35.9)	56 (37.1)	73 (35.1)
High	114 (31.8)	41 (27.2)	73 (35.1)
Individuals in the household ≥13 years, mean (95% CI)	5.6 (5.4–5.8)	5.6 (5.3–5.8)	5.6 (5.4–5.9)
Household member with diabetes, n (%)			
Household member with diabetes	149 (41.5)	99 (65.6)	111 (53.4)
No household member with diabetes	210 (58.5)	52 (34.4)	97 (46.6)
Weight status^a^, n (%)			
Underweight	44 (12.3)	19 (12.6)	25 (12.0)
Normal weight	263 (73.3)	121 (80.1)	142 (68.3)
Overweight	52 (14.5)	11 (7.3)	41 (19.7)
Smoking, n (%)			
Never smoked	288 (80.2)	114 (75.5)	174 (83.7)
Former smoker	41 (11.4)	21 (13.9)	20 (9.6)
Smoker	30 (8.4)	16 (10.6)	14 (6.7)
Alcohol intake, drinks per day	0.5 (0.3–0.8)	1.2 (0.57–1.82)	0.0 (0.0–0.0)
Aerobic capacity^b^ n (%)			
Low	121 (33.7)	55 (36.4)	66 (31.7)
Medium	155 (43.2)	59 (39.1)	96 (46.2)
High	83 (23.1)	37 (24.5)	46 (22.1)
HbA1c, mean (95% CI)	5.4 (5.4–5.5)	5.3 (5.2–5.4)	5.5 (5.4–5.5)
HbA1c n (%)			
≥ 5.7	93 (25.9)	28 (18.5)	65 (31.3)
< 5.7	266 (74.1)	123 (81.5)	143 (68.8)
Food intake^c^, mean (95% CI)	59.1 (56.8–61.4)	59.1 (55.2–63.1)	59.0 (56.2–61.9)

Data are presented as mean (95% CI) for continuous variables and as total number of participants (%) for categorical variables

*Abbreviations*: *CI* Confidence interval, *HbA1c* glycated haemoglobin, *SES* socioeconomic status

^a^Body mass index (BMI). For participants aged 19 years or younger, an sex and age specific BMI z-score (BMIZ) was calculated [18]. BMI was calculated as weight(kg)/height(m)^2^ for participants above the age of 19 years. Weight status was categorised into underweight (BMI < 18.5 or BMIZ<-1 SD), normal weight (18.5 ≤ BMI < 25 or − 1 < BMIZ<+ 1) and Overweight (BMI ≥ 25 or BMIZ> + 1)

^b^Estimated aerobic capacity (VO2-max) from 8 min step test and categorised into three categories according to Astrand [17]. Non-completion of step-test was coded as missing (4 missing)

^c^Number of food servings per week

### Weekly food intake

**Fig. 1:**
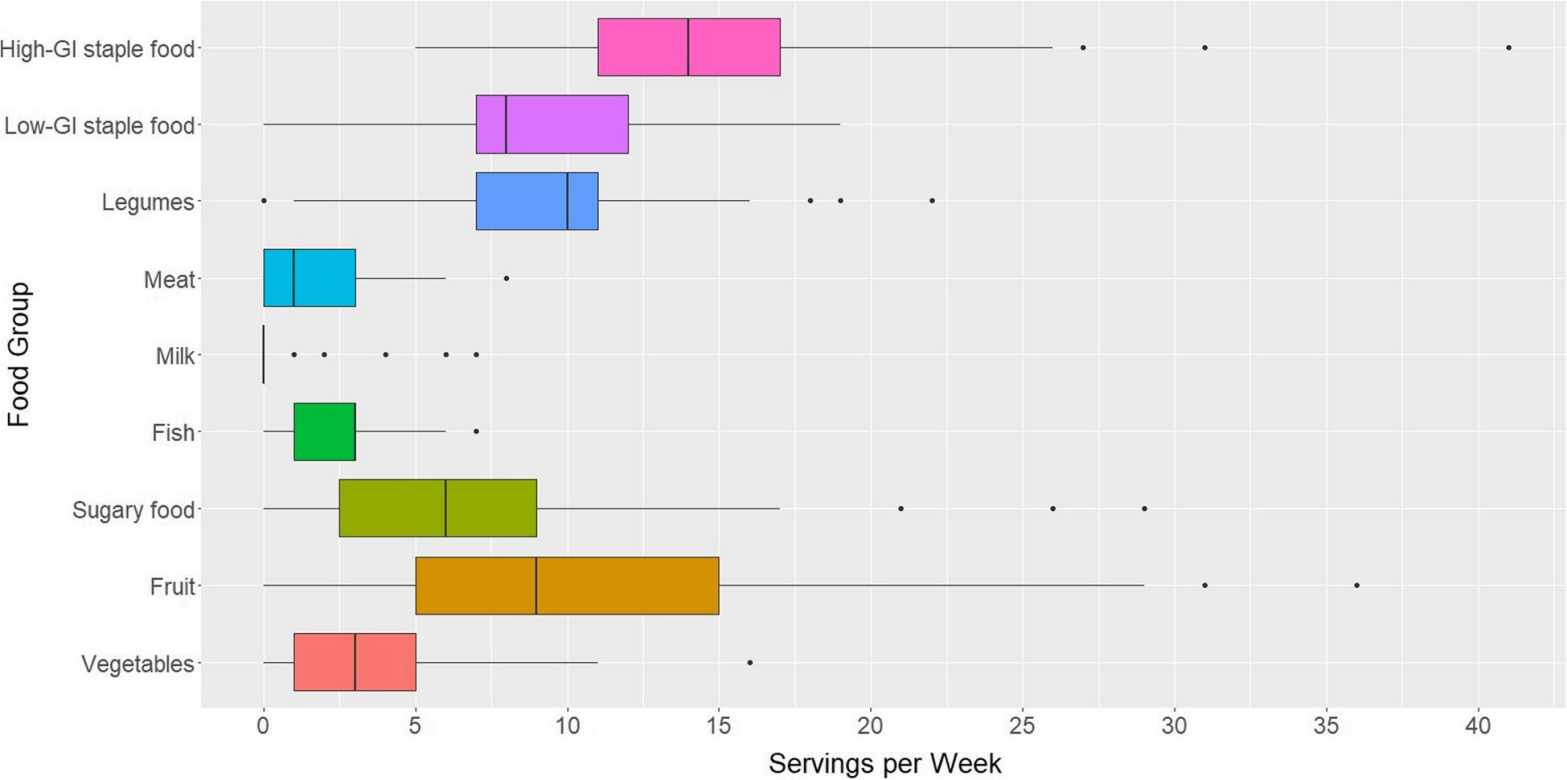
Boxplot of number of food servings/week in Kasese District, Uganda (*N* = 359), Food Intake and Cardiometabolic Risk Factors in Rural Uganda study, 2012–2013. Abbreviations: GI, glycaemic index

Median total food intake was 53 servings/week (p25-p75: 44–69). High-GI staple food was the food group with the highest median of weekly servings (14 servings/week, p25-p75: 11–17) (Fig. 1), followed by legumes (10 servings/week, p25-p75: 7–11), fruits (9 servings/week, p25-p75: 5–15) and low-GI staple food (8 servings/week, p25-p75: 7–12). In our study population, 86.1% did not consume milk at all and the highest milk intake was 7 servings/week. Median intake of meat was 1 serving/week (p25-p75: 0–3), while the median intake of fish and for vegetables was 3 servings/week (p25-p75: 1–3 for fish; p25-p75: 1–5 for vegetables). Median intake of sugary food was 6 servings/week (p25-p75: 2–9). The food items with the highest median intake in the study population was cassava (9 servings/week, p25-p75: 7–13) and plantain (7 servings/week, p25-p75: 3–7).

### Food intake and HbA1c

For total food intake, the OR of having abnormal HbA1c increased 2% (OR = 1.02, 95% CI: 1.01–1.04) with each food serving per week. Adjusting for weight status, aerobic capacity and socioeconomic status did not attenuate the association (Table 2). Intake of high-GI staple food (OR = 1.06, 95% CI: 1.00–1.12), legumes (OR = 1.10, 95% CI: 1.01–1.20), milk (OR = 1.25, 95% CI: 1.07–1.46) and sugary food (OR = 1.08, 95% CI: 1.01–1.15) were also associated with increased OR for having abnormal HbA1c. Adjustment for total food intake attenuated the association for all these food groups. (Model II, Table 2). When adjusting for total food intake (Model II), intake of fruit was associated with decreased OR for having abnormal HbA1c (OR = 0.94, 95% CI: 0.88–1.00). In the fully adjusted model (Model III, Table 2), none of the food groups were associated with decreased or increased ORs of abnormal HbA1c.

**Table 2:** Odds ratio of having abnormal glycated haemoglobin (≥ 5.7) compared to normal glycated haemoglobin (< 5.7) per increase in number of food servings/week by 1 unit in Kasese District, Uganda (*N* = 359), Food Intake and Cardiometabolic Risk Factors in Rural Uganda study, 2012–2013

Food group	Model I *N* = 359	Model II *N* = 359	Model III *N* = 359
	HbA1c ≥ 5.7 (ref = HbA1c < 5.7)	HbA1c ≥ 5.7 (ref = HbA1c < 5.7)	HbA1c ≥ 5.7 (ref = HbA1c < 5.7)
Total food intake	1.02 (1.01–1.04)	–	1.02 (1.00–1.04)
High-GI staple food (GI ≥ 70)	1.06 (1.00–1.12)	1.01 (0.96–1.07)	1.01 (0.95–1.07)
Low-GI staple food (GI < 70)	1.07 (0.99–1.15)	0.98 (0.91–1.06)	0.98 (0.90–1.06)
Legumes	1.10 (1.01–1.20)	1.04 (0.95–1.12)	1.03 (0.94–1.12)
Meat	1.20 (1.00–1.43)	1.09 (0.95–1.26)	1.08 (0.93–1.25)
Milk	1.25 (1.07–1.46)	1.14 (0.99–1.30)	1.11 (0.96–1.29)
Fish	1.09 (0.86–1.38)	1.02 (0.84–1.23)	1.00 (0.84–1.19)
Sugary food	1.08 (1.01–1.15)	1.02 (0.94–1.09)	1.02 (0.95–1.09)
Fruits	1.04 (0.99–1.09)	0.94 (0.88–1.00)	0.95 (0.89–1.02)
Vegetables	1.05 (0.94–1.18)	0.99 (0.90–1.08)	0.97 (0.88–1.07)

Values are shown as odds ratio (95% confidence interval) per food serving per week

Model 1: Adjusted for sex and age, including household as a random effect

Model 2: Model 1 + total food intake

Model 3: Model 2 + weight status, aerobic capacity and socioeconomic status

*Abbreviations*: *HbA1c* glycated haemoglobin, *GI* glycaemic index

### Food intake and BMI

Total food intake was associated with 1% (OR = 1.01, 95% CI: 1.00–1.03) increased OR of being overweight compared to normal weight per one additional serving of food per week (Table 3). Of specific food groups, intake of meat (OR = 1.38, 95% CI: 1.16–1.64) and low-GI staple food (OR = 1.09, 95% CI: 1.02–1.17) increased the OR of being overweight. Adjustment for total food intake attenuated the OR for low-GI staple food (OR = 1.05, 95% CI: 0.96–1.15), but not the association between meat intake and increased OR of overweight. The association between meat intake and overweight was not attenuated by adjustment for aerobic capacity and socioeconomic status (Model III, Table 3) (OR = 1.33, 95% CI: 1.08–1.64). We did not find any association between underweight and total food intake or specific food groups (Table 3).

**Table 3:** Odds ratio of being underweight or overweight compared to normal weight per increase in number of food servings/week by 1 unit in Kasese District, Uganda (*N* = 359), Food Intake and Cardiometabolic Risk Factors in Rural Uganda study, 2012–2013

Food group	Model I *N* = 363		Model II *N* = 359		Model III *N* = 359	
	Underweight (ref = normal weight)	Overweight (ref = normal weight)	Underweight (ref = normal weight)	Overweight (ref = normal weight)	Underweight (ref = normal weight)	Overweight (ref = normal weight)
Total food intake	0.99 (0.96–1.01)	1.01 (1.00–1.03)	–	–	0.99 (0.97–1.01)	1.01 (1.00–1.03)
High-GI staple food (GI ≥ 70)	0.98 (0.92–1.05)	1.03 (0.96–1.11)	1.00 (0.93–1.07)	0.99 (0.93–1.05)	0.99 (0.91–1.07)	1.01 (0.95–1.07)
Low-GI staple food (GI < 70)	0.98 (0.90–1.05)	1.09 (1.02–1.17)	100 (0.91–1.11)	1.06 (0.97–1.17)	1.02 (0.91–1.14)	1.05 (0.96–1.15)
Legumes	0.98 (0.91–1.05)	1.01 (0.90–1.13)	1.01 (0.92–1.10)	0.94 (0.85–1.04)	0.98 (0.90–1.06)	0.95 (0.87–1.05)
Meat	1.08 (0.91–1.27)	1.38 (1.16–1.64)	1.13 (0.95–1.36)	1.34 (1.11–1.63)	1.17 (0.99–1.39)	1.33 (1.08–1.64)
Milk	0.93 (0.73–1.18)	1.19 (0.99–1.43)	0.97 (0.76–1.23)	1.13 (0.93–1.38)	0.99 (0.77–1.27)	1.10 (0.92–1.33)
Fish	1.01 (0.84–1.22)	1.05 (0.79–1.40)	1.02 (0.85–1.23)	0.99 (0.76–1.29)	0.99 (0.82–1.21)	1.01 (0.80–1.28)
Sugary food	0.95 (0.87–1.03)	1.05 (0.98–1.13)	0.95 (0.88–1.03)	1.01 (0.92–1.11)	0.96 (0.89–1.04)	1.00 (0.91–1.09)
Fruits	0.98 (0.93–1.03)	1.03 (0.97–1.08)	1.00 (0.92–1.09)	0.96 (0.89–1.04)	1.02 (0.94–1.10)	0.96 (0.89–1.03)
Vegetables	0.99 (0.90–1.10)	1.00 (0.88–1.13)	1.02 (0.92–1.13)	0.94 (0.83–1.06)	1.01 (0.91–1.12)	0.92 (0.81–1.06)

Values are shown as odds ratio (95% confidence interval) per food serving per week

Model 1: Adjusted for sex and age, including household as a random effect

Model 2: Model 1 + total food intake

Model 3: Model 2 + aerobic capacity and socioeconomic status

*Abbreviations*: *GI* glycaemic index

## Discussion

We found that food intake in general was monotonously and dominated by staple food, especially cassava and plantain, and legumes and fruits. Our results showed that increasing number of food servings was associated with increased OR of abnormal HbA1c and being overweight. In terms of specific food groups, each weekly serving of meat was associated with a 33% increased OR of being overweight. Each weekly serving of fruit was associated with a 6% decreased OR of abnormal HbA1c, however, after further adjustment for weight status, aerobic capacity and socioeconomic status, the association was attenuated. Our findings of increased OR of abnormal HbA1c and overweight with increasing number of food servings were in accordance with previous studies showing an association between increased total energy intake and increased risk of T2D potentially mediated through overweight [22, 23].

We found that the OR of abnormal HbA1c decreased with increased number of fruit servings, which is in accordance with a meta-analysis by Wang et al. showing that a higher intake of fruit and vegetables was associated with a decreased risk of T2D [24]. We found a tendency that intake of fruit and vegetables was associated with a lower OR of overweight in the present study. Results of previous research have suggested that intake of fruit and vegetables may reduce overweight by replacing food items with high fat content [25]. WHO recommends five portions of fruit and vegetables per day (excluding staple food e.g. cassava) [6]. In the present study, median intake of fruit was 1.3 per day (approx. 17% of total food intake), while median intake of vegetables was 0.4 per day (approx. 6% of total food intake).

Results from previous studies showed an association between intake of red and processed meat and increased risk of T2D and overweight [26–28]. In the present study, we found that meat intake was associated with higher OR of overweight. However, we found no association between meat intake and abnormal HbA1c, which may be explained by a general low intake of meat in our study population (median = 1 serving per week), that the food groups in our study did not differentiate between red and white meat, and that meat intake primarily came from free-range domestic animals and not processed meat.

Approximately 11% of total food intake in our study population consisted of sugary food, which is higher than the 10% recommended by WHO [29]. A systematic review of Sonestedt et al., found an association between intake of sugar-sweetened beverages and higher risk of T2D, but no association for other types of sugary food [30]. Thus, our findings were not in accordance with previous research, as we did not find an association between intake of sugary food and HbA1c or weight status, even though the sugary food group in the present study included sugary drinks e.g. soda and juice as sugary food.

We found an association between high-GI staple food and abnormal HbA1c, which is in line with a previous meta-analysis of three large US cohorts showing an association between increased intake of high-GI staple food and risk of T2D [31]. Though, the association attenuated in the present study after adjustment for total food intake.

Intake of cereal fibres has been associated with decreased risk of developing T2D and obesity [32]. In contrast, we found that low-GI staple food was associated with increased OR of overweight in Model I, and the same trend was observed after adjustment for total food intake, which is not in line with previous studies.

In accordance with previously reported food consumption in Uganda [33], our study population had a monotonous diet with the most consumed food items being staple food; cassava and plantain. Further, intake of processed food was low, but intake of sugary foods was higher than the 10% recommended by WHO. This suggests that the nutritional transition in our rural Ugandans study population may only be in the initial phase [1]. The monotonous diet may also explain why we found little variation in intake of specific food groups between people with different HbA1c levels and weight status. However, the monotonous diet in itself may also place the study population in risk of cardiometabolic diseases, as high dietary diversity has been associated with decreased risk of T2D [34]. In addition, the number of people who were overweight or obese was low in our study population (14%). Further, the study population was younger than the average age at diagnosis of T2D in Uganda (age 52 years) [35], and generally healthy as we excluded individuals with diagnosed T2D. Lastly, many of the participants had a high activity level as they were peasants and walked long distances [36].

We did not find any associations between intake of food groups and cardiometabolic risk factors, which were not in accordance with previous studies from HIC, even though the food items that made up the food groups in our study were different from HIC settings, e.g. stable food in our study population consisted mainly of cassava and plantain while bread and potatoes are more common stables in HIC [37, 38]. The monotone diet in our study population may put the people at risk of nutritious deficiencies [39] and may be caused by lack of access to a varied diet. Thus, in this rural district around 48% lived below the poverty line, many of the participants were subsistence farmers and land size has been decreasing as land is inherited patrilineally, and due to an increasing population growth, the size of land holdings is decreasing rapidly [40, 41]. Even though cardiometabolic risk factors generally were low in the study population, the high sugar intake may indicate a nutritional transition.

### Limitations

The present study had certain strengths. This was the first study to investigate the association between food intake and cardiometabolic risk factors in a rural East African population. The study population lived in a hard-to-reach area, and the study had several methodological strengths including objective individual measures of HbA1c and BMI, a locally adapted FFQ and adjustment for possible confounders. Households including a member with T2D were selected while controls were randomly chosen with a high response rate of 90% for households and 97.5% for individuals. However, the study also had some limitations: firstly, data on food intake was reported as servings per week and collected at household level. We considered this a valid measure since the vast majority of the household members shared meals and it was rare for the study population to eat outside their home (only 1.4% of the households included family members, who reported to sometimes eat outside their home), though differences in portion sizes and number of servings between household members may have caused imprecise reporting of food intake. Secondly, the FFQ as well as the food groups used in the present study was designed to assess dietary diversity, not cardiometabolic risk factors [13]. Thirdly, the FFQ of the present study was not validated against other methods of assessing food consumption. Therefore, underreporting of certain food items, not incorporated in the FFQ, was possible. Fourth, cooking methods were not assessed in the present study, which could have caused differences in the energy content of the same food item. Missing information on specific food items and cooking method would potentially attenuate the association between food intake and cardiometabolic risk factors.

We used data from a case-control household study. Even though participants with diagnosed T2D were excluded from the present study, participants having a household member diagnosed with T2D may have had higher predisposed genetic risk of abnormal HbA1c and may have changed their diet over time according to dietary recommendations for T2D. However, additional sensitivity analysis did not show any different results, when excluding households with T2D (results not shown). Also, in a previous study in the same study population [13], we found lower HbA1c among residents below the age of 30 years in diabetic households [13].

Data were collected in 2012–2013, however, we are not aware of major political or economic changes or reforms that would have influenced agriculture, crops or eating patterns.

## Conclusion

In conclusion, we found a monotonous diet in our study population including protective components for cardiometabolic diseases, such as low consumption of meat and relatively high consumption of legumes and fruits. Despite a low meat intake, a 33% increased OR of overweight was found with each additional weekly serving of meat. Fruit intake was associated with decreased OR of abnormal HbA1c, though the association was attenuated after adjustment for weight status, aerobic capacity and socioeconomic status. Several risk factors for cardiometabolic disease was also observed in the diet, such as a relatively high consumption of sugary food, and low consumption of vegetables. Thus, the rural Ugandan population is most likely gradually transitioning towards a more unhealthy diet. In Uganda, nutrition strategies in general and those to prevent cardiometabolic diseases should focus on how people in rural areas can access and utilize existing resources sustainable e.g. fish from local lakes and grow crops that will provide both quantity, quality and a varied diet.

## Supplementary Information

**Additional file 1.**

**Additional file 2: Table 1.** Food items included in food groups in Kasese District, Uganda (*N*=359), Food Intake and Cardiometabolic Risk Factors in Rural Uganda study, 2012–2013

## Data Availability

Not applicable.

## Abbreviations

VO2-max: Aerobic capacity
BMI: Body mass index
BMIZ: BMI z-score
CI: Confidence interval
FAO: Food and Agriculture Organization of the United Nations
FFQ: Food frequency questionnaire
GI: Glycaemic Index
HbA1c: Glycated haemoglobin
HIC: High income countries
LMIC: Low- and middle income countries
NCD: Non-communicable diseases
OR: Odds Ratio
SES: Socioeconomic status
SSA: Sub-Sahara Africa
T2D: Type 2 diabetes
WHO: World Health Organization

## References

[1] Popkin BM (2006). Global nutrition dynamics: the world is shifting rapidly toward a diet linked with noncommunicable diseases. Am J Clin Nutr.

[2] Omran AR (1971). The epidemiologic Transiton - a theory of the epidemiology of population change. Milbank Meml Fund Quaterly.

[3] Maher D, Smeeth L, Sekajugo J (2010). Health transition in Africa: practical policy proposals for primary care. Bull World Health Organ.

[4] Food and Agriculture Organization of the United Nations (2010). Uganda. Nutrition country profile.

[5] Afshin A, Sur PJ, Fay KA, Cornaby L, Ferrara G, Salama JS (2019). Health effects of dietary risks in 195 countries, 1990–2017: a systematic analysis for the global burden of disease study 2017. Lancet..

[6] World Health Organization (2018). Healthy diet. Fact sheets.

[7] Mwenda V, Mwangi M, Nyanjau L, Gichu M, Kyobutungi C, Kibachio J. Dietary risk factors for non-communicable diseases in Kenya: findings of the STEPS survey, 2015. BMC Public Health. 2018;18(S3):97–104. 10.1186/s12889-018-6060-y PMC6219002 30400904

[8] Otiso KM (2006). Cuisine and traditional dress. Culture and customs of Uganda (cultures and customs of the world).

[9] Bahendeka S, Wesonga R, Mutungi G, Muwonge J, Neema S, Guwatudde D (2016). Prevalence and correlates of diabetes mellitus in Uganda: a population-based national survey. Tropical Med Int Health.

[10] International Diabetes Federation (2017). IDF Diabetes Atlas.

[11] Yaya S, Ghose B. Trend in overweight and obesity among women of reproductive age in Uganda: 1995-2016. Obes Sci Pract. 2019;5(4):312–23. 10.1002/osp4.351 PMC6700515 31452916

[12] Kirunda BE, Fadnes LT, Wamani H, Van Den Broeck J, Tylleskär T (2015). Population-based survey of overweight and obesity and the associated factors in peri-urban and rural eastern Uganda chronic disease epidemiology. BMC Public Health.

[13] Nielsen J, Bahendeka SK, Gregg EW, Whyte SR, Bygbjerg IC, Meyrowitsch DW (2015). A comparison of cardiometabolic risk factors in households in rural Uganda with and without a resident with type 2 diabetes, 2012-2013. Prev Chronic Dis.

[14] Nielsen J. Living with type 2 diabetes in rural Uganda - exploring the household as an intersection for diabetes management, risks, and behaviors [dissertation]. Copenhagen: University of Copenhagen; 2014.

[15] Kennedy G, Ballard T, Dop M (2011). Guidelines for measuring household and individual dietary diversity.

[16] Atkinson F, Foster-Powell K, Brand-Miller JC (2008). Glycemic load values : 2008. Diabetes Care.

[17] American Diabetes Association (2020). Understanding A1C.

[18] de Onis M, Onyango AW, Borghi E, Siyam A, Siekmann J (2007). Development of a WHO growth reference for school-aged children and adolescents. Bull World Health Organ.

[19] World Health Organization (2019). BMI Classification. Gloabl Database on Body Mass Index.

[20] Astrand I (1960). Aerobic work capacity in men and women with special reference to age. Acta Physiol Scand Suppl.

[21] Vyas S, Kumaranayake L (2006). Constructing socio-economic status indices: how to use principal components analysis. Health Policy Plan.

[22] Villegas R, Shu XO, Yang G, Matthews CE, Li H, Cai H (2008). Energy balance and type 2 diabetes: a report from the Shanghai Women’s health study. Nutr Metab Cardiovasc Dis.

[23] Tinker LF, Sarto GE, Howard BV, Huang Y, Neuhouser ML, Mossavar-Rahmani Y (2011). Biomarker-calibrated dietary energy and protein intake associations with diabetes risk among postmenopausal women from the Women’s health initiative. Am J Clin Nutr.

[24] Wang PY, Fang JC, Gao ZH, Zhang C, Xie SY (2016). Higher intake of fruits, vegetables or their fiber reduces the risk of type 2 diabetes: a meta-analysis. J Diabetes Investig.

[25] Boeing H, Bechthold A, Bub A, Ellinger S, Haller D, Kroke A (2012). Critical review: vegetables and fruit in the prevention of chronic diseases. Eur J Nutr.

[26] Pan A, Sun Q, Bernstein A (2011). Red meat consumption and risk of type 2 diabetes: 3 cohorts of US adults and an updated meta-analysis. Am J Clin Nutr.

[27] Song Y, Manson JE, Buring JE, Liu S (2004). A prospective study of red meat consumption and type 2 diabetes in middle-aged and elderly women: the women’s health study. Diabetes Care.

[28] Rouhani MH, Salehi-Abargouei A, Surkan PJ, Azadbakht L (2014). Is there a relationship between red or processed meat intake and obesity? A systematic review and meta-analysis of observational studies. Obes Rev.

[29] WHO (2018). Healthy diet. Fact sheet.

[30] Sonestedt E, Øverby N, Laaksonen D, Eva BB (2012). Does high sugar consumption exacerbate cardiometabolic risk factors and increase the risk of type 2 diabetes and cardiovascular disease?. Food Nutr Res.

[31] Bhupathiraju SN, Tobias DK, Malik VS, Pan A, Hruby A, Manson JE (2014). Glycemic index, glycemic load, and risk of type 2 diabetes: results from 3 large US cohorts and an updated meta-analysis. Am J Clin Nutr.

[32] Priebe M, van Binsbergen J, de Vos R, Vonk RJ. Whole grain foods for the prevention of type 2 diabetes mellitus. Cochrane Database Syst Rev. 2008;(1):CD006061. 10.1002/14651858.CD006061.pub2 PMC10384032 18254091

[33] Harvey P, Rambeloson Z, Dary O (2010). The 2008 Uganda Food Consumption Survey: Determining the Dietary Patterns of Ugandan Women and Children. A2Z: The USAID Micronutrient and Child Blindness Project, AED, Washington D.C.

[34] Conklin AI, Monsivais P, Khaw KT, Wareham NJ, Forouhi NG (2016). Dietary diversity, diet cost, and incidence of type 2 diabetes in the United Kingdom: a prospective cohort study. PLoS Med.

[35] Muddu M, Mutebi E, Ssinabulya I, Kizito S, Mondo CK (2018). Hypertension among newly diagnosed diabetic patients at Mulago National Referral Hospital in Uganda: a cross sectional study. Cardiovasc J Afr.

[36] Nielsen J, Bahendeka SK, Bygbjerg IC, Meyrowitsch DW, Whyte SR (2016). Diabetes treatment as “homework”: consequences for household knowledge and health practices in rural Uganda. Health Educ Behav.

[37] Heidemann C, Hoffmann K, Spranger J, Klipstein-Grobusch K, Möhlig M, Pfeiffer AFH (2005). A dietary pattern protective against type 2 diabetes in the European prospective investigation into Cancer and nutrition (EPIC) - Potsdam study cohort. Diabetologia..

[38] van Dam RM, Rimm EB, Willett WC, Stampfer MJ, Hu FB (2002). Dietary patterns and risk for type 2 diabetes mellitus in U.S. Ann Intern Med.

[39] Joint WHO / FAO Expert Consultation. Diet, nutrition and the prevention of chronic diseases. Geneva; 2003. Available from: https://apps.who.int/iris/bitstream/handle/10665/42665/WHO_TRS_916.pdf?sequence=1. Accessed 21 Oct 2020.

[40] Uganda Bureau of Statistics. Kasese District local government statistical abstract. Kasese: Kasese District Local Government; 2012.

[41] Renno DC, Twinamasiko J, Mugisa CP. Kasese District poverty profiling and mapping 2011–2012. Kasese District Local Government, Planning Unit and the Belgian Technical Cooperation. Kasese: Kasese District Poverty Reduction Programme; 2012.

