# Supplementary material for: Food intake and cardiometabolic risk factors in rural Uganda
Source: Arch Public Health. 2021 Feb 25;79:24. doi: 10.1186/s13690-021-00547-x (PMC7908644; doi:10.1186/s13690-021-00547-x)
Supplement: Supplementary file 1 — Additional file 1. [file 13690_2021_547_MOESM1_ESM.docx]

Additional file 1: Holmager et al. Food Intake and Cardiometabolic Risk Factors in Rural Uganda

Non-diabetic households were selected randomly by facing the front of the house belonging to a diabetic household, then walking leftwards and visiting the fifth compound. If this household included an individual with an age in the range +/- five years of the person with T2D from the diabetic household and the household fulfilled the inclusion criteria, the household members were invited to participate in the study. If no person within this age-range lived in the household or the household did not fulfill the inclusion criteria, the next compound to the left was visited (5+1). In addition to fulfilling the same inclusion and exclusion criteria as the diabetic households, should have no individuals with diagnosed diabetes. Only household members who were ≥ 13 years of age and who had lived in the households for at least three months prior to the first visit by the study team were included in the study.

Sample size was initially calculated to investigate difference in plasma glucose for individuals without previously diagnosed T2D in non-diabetic and diabetic households, respectively. Individuals without previously diagnosed T2D in diabetic household was expected to have 10% higher plasma glucose due to an increased genetically predisposition for T2D. A previous study, showed that the average plasma glucose level for a rural Ugandan without previously diagnosed was 4.8 mmol/l [1], thus, the difference was expected to be 0.48 mmol/l. To detect a difference with a power of 80% and α=0.05 a sample size of 232 participants should be included. However, intra-class correlation in genetic predisposition was expected within households, though, the intra-class correlation was not expected to go above 0.15. Assuming an average of four household members the sample size was calculated to be 232 x 1.45 = 337 individuals and 337 / 4 = 85 households. Consequently, 90 households (45 diabetic and 45 non-diabetic) was included in the study.

**References**

1. Maher D, Waswa L, Baisley K, Karabarinde A, Unwin N, Grosskurth H. Distribution of hyperglycaemia and related cardiovascular disease risk factors in low-income countries: A cross-sectional population-based survey in rural Uganda. Int J Epidemiol. 2011;40:160–71.
