# Supplementary material for: Food intake and cardiometabolic risk factors in rural Uganda
Source: Arch Public Health. 2021 Feb 25;79:24. doi: 10.1186/s13690-021-00547-x (PMC7908644; doi:10.1186/s13690-021-00547-x)
Supplement: Supplementary file 2 — Additional file 2: Table 1. Food items included in food groups in Kasese District, Uganda (N=359), Food Intake and Cardiometabolic Risk Factors in Rural Uganda study, 2012–2013 [file 13690_2021_547_MOESM2_ESM.docx]

Additional file 2: Holmager et al. Food Intake and Cardiometabolic Risk Factors in Rural Uganda

**Table 1** Food items included in food groups in Kasese District, Uganda (N=359), Food Intake and Cardiometabolic Risk Factors in Rural Uganda study, 2012-2013

| High-GI staple food (GI≥70) | Bread, rice, posho (maize dough), maize porridge, white potatoes, cassava, ubundu (cassava dough), pancake, mandazi |
| --- | --- |
| Low-GI staple food (GI<70) | Yams, sweet potatoes, matoke (green plantain), pumpkin, spaghetti, millet porridge, chapatti, fried potatoes |
| Legumes | Beans, fried beans, groundnuts, groundnut sauce, soybean milk |
| Meat | Duck stew, chicken stew, goat stew, sheep stew, pork stew, cow stew |
| Milk | Cow Milk |
| Fish | Fish stew |
| Sweets | Sugarcane, honey, biscuit, sweets, soda, tea with sugar, juice |
| Fruits | Pinapple, papaya, avocado, banana, jack fruit, ebidadayimu (local fruit), passion fruit, watermelon, guava, mango, apple, lemon, orange, coconut |
| Vegetables | Fried eggplant, fried cabbage, vegetable sauce |

Abbreviations: GI, glycaemic index.
